# Development of a High-Efficiency Immunomagnetic Enrichment Method for Detection of Human Norovirus via PAMAM Dendrimer/SA-Biotin Mediated Cascade-Amplification

**DOI:** 10.3389/fmicb.2021.673872

**Published:** 2021-07-20

**Authors:** Junshan Gao, Le Zhang, Liang Xue, Weicheng Cai, Zhiwei Qin, Jiale Yang, Yanhui Liang, Linping Wang, Moutong Chen, Qinghua Ye, Ying Li, Juan Wang, Shi Wu, Qingping Wu, Jumei Zhang

**Affiliations:** ^1^Guangdong Provincial Key Laboratory of Microbial Safety and Health, State Key Laboratory of Applied Microbiology Southern China, Institute of Microbiology, Guangdong Academy of Sciences, Guangzhou, China; ^2^College of Food Science, South China Agricultural University, Guangzhou, China

**Keywords:** noroviruses, oysters, PAMAM-mediated, immunomagnetic enrichment, biotin amplification

## Abstract

Human norovirus is a common cause of acute gastroenteritis worldwide, and oysters have been found to be the main carriers for its spread. The lack of efficient pre-treatment methods has been a major bottleneck limiting the detection of viruses in oysters. In this study, we established a novel immunomagnetic enrichment method using polyamidoamine (PAMAM) dendrimer/SA-biotin-mediated cascade amplification for reverse transcriptase quantitative real-time polymerase chain reaction (RT-qPCR) detection. We compared the capture efficiency of traditional immunomagnetic enrichment, biotin-amplified immunomagnetic enrichment, and PAMAM dendrimer/SA-biotin-mediated cascade-amplification immunomagnetic enrichment. The optimal capture efficiency of the novel method was 44.26 ± 1.45%, which increased by 183.17% (*P* < 0.01) and 18.09% (*P* < 0.05) compared with the first two methods, respectively. Three methods were all applied in detecting norovirus in 44 retail oysters, the detection rate of the PAMAM dendrimer/SA-biotin-mediated method was 25.0%, which was higher than those of traditional IME (15.90%) and SA-biotin-amplified IME (18.80%) by 9.1 and 6.2%, respectively. In conclusion, the novel method can be applied for the rapid detection of norovirus in oysters, which can help reduce the cost and time of detection and improve detection rates.

## Introduction

Norovirus (NoV) is one of the main causes of acute gastroenteritis (Ahmed et al., 2014) and frequently appears in closed spaces in hospitals, schools, and cruise ships (Siebenga et al., 2009). The consumption of bivalve shellfish, such as oysters, is a common route of NoV infection (Woods et al., 2016; Le Mennec et al., 2017). Oysters are filter feeders, and NoV particles easily accumulate in their digestive tract (Le Guyader et al., 2012). As oysters are usually eaten raw, this increases the rate of NoV transmission (El Moqri et al., 2019).

Accurate diagnosis of NoV contamination in oysters is essential to control NoV outbreaks and to ensure that patients receive optimal treatment. Real time RT-PCR (RT-qPCR) is the “gold standard” for NoV diagnosis and detection (Vinjé, 2015). However, this method is often affected by low amounts of NoVs and by presence of inhibitors in the sample, such as polysaccharides, lipids, and proteins in oyster tissue. Therefore, it is necessary to establish an efficient NoV enrichment method to minimize the influence of the inhibitors. Traditional enrichment methods include direct treatment, proteinase K digestion and proteinase K digestion combined with polyethylene glycol precipitation (Lee and Lee, 1981; Wu et al., 2016). However, these methods are non-specific and cannot fully remove the interference of complex matrices.

Currently, immunomagnetic enrichment (IME) is widely used to separate and concentrate pathogens from food samples in a more effective way than traditional methods (Valdes et al., 2009; Xiong et al., 2014). Traditional IME combines magnetic beads with antibodies to enrich the target from a complex matrix (Yao et al., 2009), and covalent coupling is commonly used to immobilize antibodies on magnetic beads (Cao et al., 2012). Covalent coupling is simple and effective, but the system is easily affected by small changes in the pH and cross-linking time. The streptavidin (SA)-biotin mediated IME has gained widespread applications because of its high affinity and specificity. SA is a tetrameric protein composed of identical subunits. One streptavidin molecule can bind to four biotin molecules to achieve signal amplification (Yuan et al., 2000).

**FIGURE 1:**
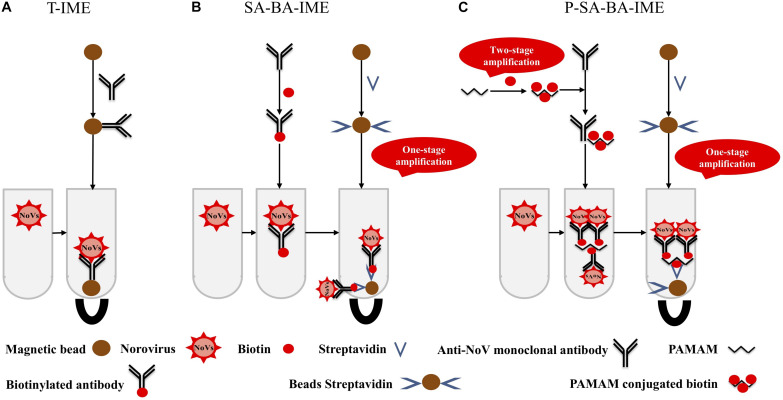
Schematic illustration of **(A)** traditional immunomagnetic enrichment (T-IME), **(B)** streptavidin-biotin amplified IME (SA-BA-IME), and **(C)** polyamidoamine (PAMAM) dendrimer/SA-biotin mediated cascade-amplification IME (P-SA-BA-IME). In the T-IME method, antibodies are directly coupled on the surface of magnetic beads by covalent bonds to prepare immunomagnetic beads. In the SA-BA-IME system, the antibody is labeled with biotin to enrich the norovirus concentration in the sample, and then forms an immunomagnetic bead complex with streptavidin magnetic nanoparticles. Based on the P-SA-BA-IME, PAMAM is selected as the coupling carrier of biotin. The surface of PAMAM contains a large number of amino sites to perform the amplification of biotin.

Polyamidoamine (PAMAM) is a dendritic polymer with a large number of functional groups available for surface modifications such as acetylation and glycosylation (Yellepeddi et al., 2009). PAMAM has good biocompatibility and no immunogenicity, it can be used as a coupling carrier for drugs and genes, and has good application prospects in the fields of biology and medicine. The molecular surface of PAMAM has a large number of positively charged amino groups, which can be combined with biotin to amplify the biotin signal. This makes a PAMAM-biotin monoclonal antibody (mAb) molecule capture more NoVs than SA-biotin mAb, increasing the capture efficiency (CE) of the enrichment process (Figure 1). In this study, cascade signal amplification was achieved by PAMAM as the coupling carrier for biotin. To the best of our knowledge, there are no reports on PAMAM as a carrier for cascade-amplification for NoV enrichment in oysters. We aimed to establish a sensitive and efficient PAMAM dendrimer/SA-biotin-mediated cascade-amplification IME (P-SA-BA-IME) method, which was combined with RT-qPCR to detect NoVs in oysters.

## Materials and Methods

### NoV Stool Samples, Oyster Samples and Anti-NoV mAb

NoV-positive stool samples used in this study were collected from The Third Affiliated Hospital of Sun Yat-sen University in our previous works and stored at −80°C in our laboratory (Xue et al., 2013, 2016, 2019). In brief, the stools samples were diluted using diethyl pyrocarbonate (DEPC)-treated phosphate buffered saline (PBS) solution to 10–20% (m/v), and then centrifuged at 7,000 × g for 5 min. The supernatant was stored as the viral stock at −80°C. The sample was identified as GII.4 by one-step RT-PCR and sequencing (Xue et al., 2013, 2016, 2019). Before use, the virus supernatant was taken out to extract total RNA, and the titer was determined by RT-qPCR as 6.24 × 10^5^ copies/μL. The appropriate titers used in this study were obtained by dilution with DEPC-treated PBS (Zhang et al., 2020). Oysters were obtained from Huangsha Aquatic Product Trading Market in Guangzhou. The anti-NoV mAb was prepared by immunizing P particles of GII.4 NoV in our previous experiment, it could react with GII.2, GII.3, GII.4, GII.6, GII.17 NoVs and be used in enzyme linked immunosorbent assay (ELISA) and colloidal gold immunochromatographic assay (Gao et al., 2021).

### Processing of the Oyster Samples

The oysters were dissected, and the digestive glands were removed and grounded to a homogenized state. One milliliter PBS and 1.5 g of oyster sample were mixed, centrifuged at 3,000 × *g* at 4°C for 10 min, and the supernatant was transferred to another clean tube for enrichment (When comparing the detection rates of the three methods, the supernatant of 4.5 g digestive glands was divided into three evenly). Oyster negative sample and oyster positive sample were used as a negative control and a positive control to avoid cross-contamination, they were tested by the P-SA-BA-IME method and a modified ISO/TS 15216-2:2013 standard method which was based on the protease K method with the increasing extraction buffer volume and PEG precipitation (Zhang et al., 2020).

### Preparation of the Traditional Immunomagnetic Beads (IMBs)

Nanomagnetic beads (750 nm) (Aorun Micronano New Material Technology Company, Shanghai, China) were washed with morpholine ethanesulfonic acid (Sigma Aldrich, St. Louis, MO, United States) three times at a volume ratio of 1:10 to ensure monodispersion, then 24 μL 1- (3-Dimethylaminopropyl)-3-ethylcarbodiimide hydrochloride (EDC-HCl) (200 mM) (Thermo Fisher Scientific Inc., Rockford, United States) and 240 μL N-hydroxysulfosuccinimide (Sulfo-NHS) (200 mM) (Thermo Fisher Scientific Inc.) were added to 1 mg magnetic beads. The magnetic beads were mixed with 40 μg monoclonal antibody for 30 min at 37°C, then 1 mL 1% (w/v) BSA (Sigma Aldrich, St. Louis, MO, United States) blocking buffer was added at 37°C for 2 h and stored at 4°C.

### Preparation of Biotinylated Monoclonal Antibody (Biotin-mAb)

The mAb was mixed with sulfo-NHS-LC-biotin (Thermo Fisher Scientific Inc.) at a volume ratio of 1:20 and placed on a dynabeads MX mixer (Thermo Fisher Scientific Inc.) at 15 rpm at room temperature for 30 min. The biotin-mAb was centrifuged in a 30 KDa ultrafiltration tube (Millipore, MWCo, 30000) (Millipore, Carrigtwohill, County Cork, Ireland) at 6,000 × *g* for 10 min to remove unbound biotin, and washed four times with PBS with 0.05% Tween 20 (PBST) (0.01 M, pH 7.4).

### Preparation of Biotin-PAMAM-mAb

PAMAM (Chenyuan Organic Silicon New Material Co., Ltd., Shandong, China) was dissolved in a pH 9.0 PBS solution for a final concentration of 10 mg/mL, then mixed 1.6 mg of sulfo-NHS-LC-biotin with 1 mL PAMAM on a rotary mixer for 2 h. The unbound sulfo-NHS-LC-biotin was removed using an ultrafiltration tube, and 2-iminosulfane hydrochloride (Traut’s reagent) (Sigma Aldrich) at a final concentration of 1 mg/mL under nitrogen for 1 h to obtain biotin-PAMAM-SH. Sulfo-SMCC (Sigma Aldrich) and anti-NoV mAb were mixed at a ratio of 10:1 and incubated for 2 h at room temperature. The unbound sulfo-SMCC was removed using an ultrafiltration tube. SMCC-mAb and biotin-PAMAM-SH were mixed at a ratio of 10:1 and incubated overnight at room temperature, and then N-ethylmaleimide (Sigma Aldrich) was used to block unbound sulfhydryl groups.

### Pre-treatment Methods

Three NoV enrichment methods were compared: traditional IME (T-IME), SA-biotin-amplified IME (SA-BA-IME), and P-SA-BA-IME (Figure 1).

#### T-IME

Anti-NoV mAb-conjugated magnetic beads were added to 1 mL PBS containing 1 × 10^6^ copies of NoVs or the supernatant of 1.5 g oyster homogenate to enrich at room temperature. The IMBs were placed on a magnetic stand to remove the supernatant, resuspended and shaked with 1 mL 0.01 M PBST (pH 7.4) washing buffer, then placed on the magnetic stand to remove the supernatant for 3 times. Finally, the IMBs were resuspended in 140 μL PBS buffer, and RNA was extracted and stored at −80°C.

#### SA-BA-IME

One milliliter of PBS containing 1 × 10^6^ copies of NoVs or the supernatant of 1.5 g oyster homogenate with biotin-mAb was placed on the dynabeads MX mixer, then SA nanomagnetic beads (750 nm) (Aorun Micronano New Material Technology Company) were added and placed on the dynabeads MX mixer at 15 rpm at room temperature. The mixture was placed on a magnetic stand for magnetic separation and washed three times with 1 mL of PBST. The IMBs were resuspended in 140 μL PBS, and RNA was extracted and stored at −80°C.

#### P-SA-BA-IME

The procedure was the same as SA-BA-IME, with only biotin-mAb being replaced by biotin-PAMAM-mAb.

### RNA Extraction

Viral RNA was extracted from 140 μL IMBs suspension after enriching the NoVs with a High Pure Viral RNA Mini Kit (Magen, Guangzhou, China) following the manufacturer’ s instructions. After repeated washing to remove impurities, 50 μL of eluate was added to the column to elute the RNA.

### RT-qPCR Analysis

We quantitated enriched NoVs by RT-qPCR (Jothikumar et al., 2005) using a LightCycler^®^ 96 System (Roche, Basel, Switzerland) using a RNA PrimeScript^TM^ One-Step Quantitative RT-PCR System (TaKaRa, Dalian, China) and 2 μL of the template was added in a total reaction volume of 20 μL. The primers and probes for NoVs included QNIF2d (5′ ATG TTC AGR TGG ATG AGR TTC TCW GA 3′), COG2R (5-′TCG ACG CCA TCT TCA TTC ACA 3′), and QNIFs (5’ FAM-AGC ACG TGG GAG GGG ATC G-TAMRAc). Cycling times and temperatures were 42°C for 5 min and 95°C for 10 min, followed by 45 cycles of 95°C for 5 s and 60°C for 20 s. A standard virus titer curve was generated by extracting viral RNA from serially diluted (10^–1^–10^–7^) virus stock. RT-qPCR was performed, and the resulting cycle threshold (Ct) values were plotted against their respective dilutions. The highest measurable Ct was assigned the value of one RT-PCR unit, and a logarithmic trend line was plotted (*R*^2^ = 0.999). The copy numbers of positive and experimental samples were calculated using the standard curve equation. The samples showed a characteristic sigmoidal curve with a Ct-value < 43 were regarded as positive (> one viral genomic copy), the number of genome copies per gram of the oyster digestive gland tissue is less than 25 as negative.

### Calculation of Capture Efficiency

Capture efficiency (CE) was defined as the percentage of NoVs enriched by IMBs to the total number of NoVs. The CE (%) was calculated according to the following equation: CE (%) = C_1_/C_0_ × 100%, C1: NoV genomic copy after enrichment, and C0: NoV genomic copy before enrichment.

### Statistical Analysis

Statistical analyses were performed with Graphpad Prism 8.0.1. The CE of three methods were compared by using One-way ANOVA. Three independent experiments were conducted to determine assay consistency. The detection rate of three methods were compared through Chi-square test. For all analyses, significantly different was set as a *P*-value less than 0.05.

## Results

### T-IME Optimization

**FIGURE 2:**
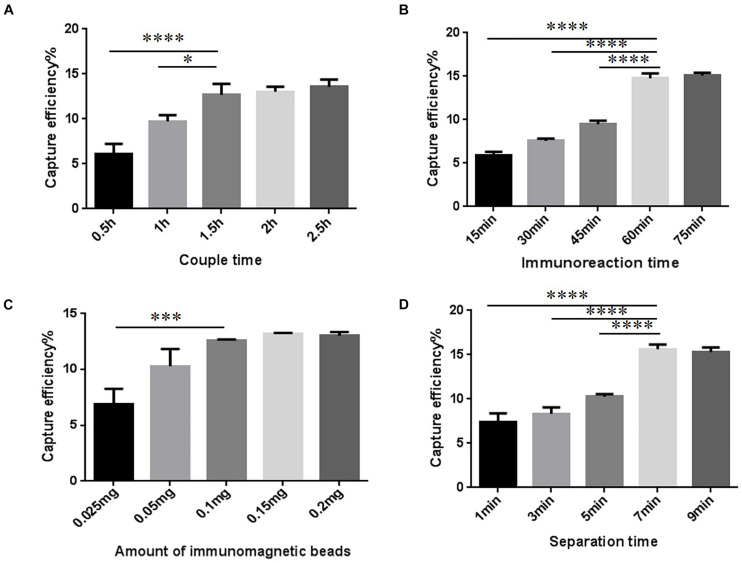
Optimization of traditional immunomagnetic enrichment (T-IME) conditions. **(A)** Optimization of couple time. **(B)** Optimization of immunoreaction time. **(C)** Optimization of the amount of immunomagnetic beads. **(D)** Optimization of separation time. Optimization of the conditions of the T-IME method by setting a series of gradients. Capture efficiency (CE) under different conditions are calculated, and the best enrichment conditions are selected based on the principle of high CE, and time and cost saving. ^∗^*P* < 0.05, ^∗∗∗^*P* < 0.001, ^****^P < 0.0001.

The conditions to increase CE of the T-IME were optimized (Figure 2A and Supplementary Table 1). When the coupling time was 30, 60, and 90 min, the CE increased to 12.68 ± 1.00%, and longer coupling times did not increase it any further. IMBs were incubated during five different times (15, 30, 45, 60, and 75 min). The optimal enrichment time was 60 min, resulting in a nearly constant CE in prolonged times (Figure 2B and Supplementary Table 2).

The amount of IMB was also optimized (Figure 2C and Supplementary Table 3). We tested different amounts of magnetic beads: 0.1, 0.15, and 0.2 mg. However, the CE was nearly stable at all quantities. Therefore, 0.1 mg of IMB was selected as the optimum amount of IMBs. The result of the separation time is shown in Figure 2D and Supplementary Table 4. When the magnetic separation time was 7 min, the CE increased to 15.63 ± 0.43%, and longer separation times did not increase CE any further.

### SA-BA-IME Optimization

**FIGURE 3:**
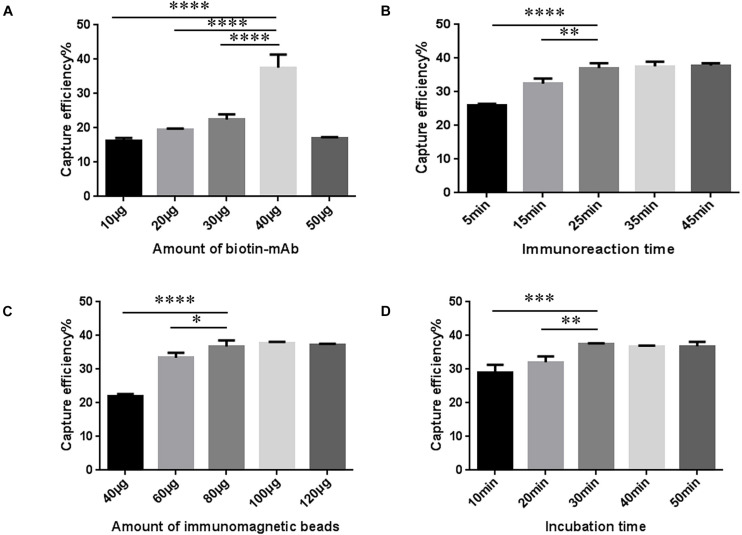
Optimization of streptavidin-biotin amplified immunomagnetic enrichment (SA-BA-IME) conditions. **(A)** Amount of biotinylated monoclonal antibody (biotin-mAb) optimization. **(B)** Optimization of immunoreaction time of biotin-mAb with noroviruses. **(C)** Optimization of the amount of immunomagnetic beads. **(D)** Optimization of incubation time. Optimization of the amount of biotin-mAb, immunoreaction time, the amount of immunomagnetic beads, and incubation time that affect the capture efficiency (CE) of the SA-BA-IME. The best enrichment conditions are selected based on the principle of high CE, and time and cost saving. ^∗^*P* < 0.05, ^∗∗^*P* < 0.01, ^∗∗∗^*P* < 0.001, ^****^P < 0.0001.

The amount of biotin mAb was optimized. When the amount of biotin-mAb was 40 μg, CE was the highest. Application of more than 40 μg biotin-mAb resulted in a sharp drop in CE (Figure 3A and Supplementary Table 5). To calculate the optimal immunoreaction time we used five different incubation times (5, 15, 25, 35, and 45 min). When the immunoreaction time reached 25 min, CE was stable (Figure 3B and Supplementary Table 6).

The results of the amount of SA magnetic beads are shown in Figure 3C and Supplementary Table 7. When the amount of SA magnetic beads was 40–80 μg, the CE increased from 22.07 ± 0.43% to 36.62 ± 1.43%, and then tended to stabilize with higher amounts. The incubation time was prolonged to 30 min, which was selected as the incubation time, as the CE of IME increased to 37.48 ± 0.20% (Figure 3D and Supplementary Table 8).

### P-SA-BA-IME Optimization

**FIGURE 4:**
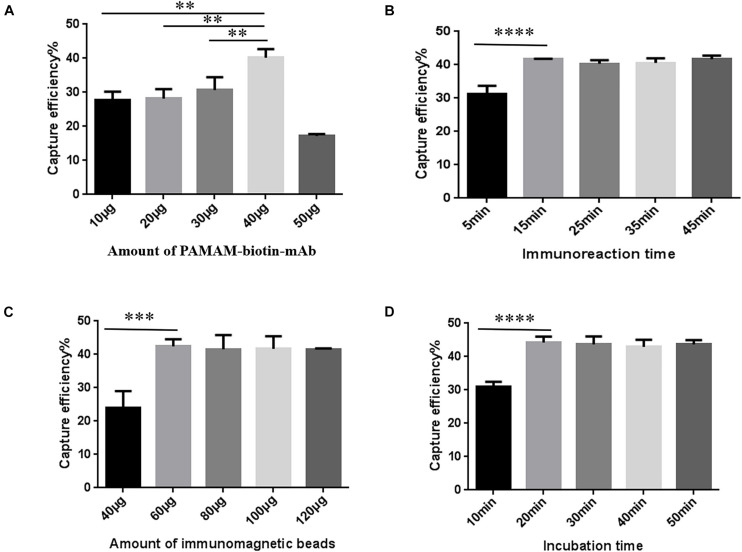
Optimization of Polyamidoamine (PAMAM) dendrimer/SA-biotin mediated cascade-amplification IME (P-SA-BA-IME) conditions. **(A)** Amount of biotin-PAMAM- monoclonal antibody (mAb) optimization. **(B)** Optimization of immunoreaction time of biotin-PAMAM-mAb with noroviruses. **(C)** Optimization of the amount of immunomagnetic beads. **(D)** Optimization of incubation time. Optimization of the conditions of P-SA-BA-IME, the best condition according to the capture efficiency under different conditions is selected based on the principle of high capture efficiency, and time and cost saving. ^∗∗^*P* < 0.01, ^∗∗∗^*P* < 0.001, ^****^P < 0.0001.

P-SA-BA-IME conditions were the same as for SA-BA-IME optimization except that biotin-mAb was replaced with biotin-PAMAM-mAb. The results are presented in Figure 4. When we increased the amount of biotin-PAMAM-mAb from 40 to 50 μg, the CE of NoVs dropped from 40.34 ± 2.04% to 17.18 ± 0.57%. Therefore, the optimal amount of biotin-PAMAM-mAb was 40 μg (Figure 4A and Supplementary Table 9). The optimal immunoreaction time for CE was 15 min, after that there was no increasing effect (Figure 4B and Supplementary Table 10). The optimal amount of SA magnetic beads was 60 μg (Figure 4C and Supplementary Table 11), and the best incubation time was 20 min (Figure 4D and Supplementary Table 12). Under these optimized conditions, the CE was 44.26 ± 1.45%. Compared with the above T-IME (15.63 ± 0.43%) and SA-BA-IME (37.48 ± 0.20%), the CE of P-SA-BA-IME were increased by 183.17% (*P* < 0.01) and 18.09% (*P* < 0.05), respectively.

### Application of Novel Methods in NoVs Detection in Retail Oysters

The three methods were applied for NoV detection in retail oyster samples (*n* = 44), and all NoV-positive results were shown in Table 1. The detection rate of P-SA-BA-IME was higher than that of the other two methods. The detection rate of T-IME was 15.90% (7/44), of SA-BA-IME was 18.80% (8/44), and of P-SA-BA-IME was 25% (11/44). There is no significant difference in the detection rates of the three methods (*P* > 0.05). One sample tested positive by T-IME, but negative by SA-BA-IME and P-SA-BA-IME; One sample tested positive by SA-BA-IME, but negative by T-IME and P-SA-BA-IME.

**TABLE 1:** NoVs-positive oysters detected by three novel methods.

Oyster samples	T-IME (genome copies/g tissue)*	SA-BA-IME (genome copies/g tissue)*	P-SA-BA-IME (genome copies/g tissue)*
1902-3	–	9.8 × 10^1^	–
1905-3	–	4.4 × 10^1^	8.0 × 10^1^
1908-5	–	–	9.8 × 10^1^
1909-1	–	–	3.5 × 10^4^
1909-2	6.7 × 10^3^	5.0 × 10^3^	1.1 × 10^3^
1910-1	1.2 × 10^2^	–	–
1910-4	–	–	9.1 × 10^2^
1910-5	3.8 × 10^3^	2.2 × 10^3^	5.5 × 10^3^
1911-1	4.8 × 10^3^	4.3 × 10^3^	9.4 × 10^4^
1911-2	1.1 × 10^4^	–	3.2 × 10^3^
1911-3	–	2.4 × 10^2^	3.3 × 10^2^
1911-4	3.8 × 10^2^	1.9 × 10^2^	1.5 × 10^2^
1911-5	1.2 × 10^2^	1.8 × 10^2^	1.3 × 10^2^
Total number	7.0 × 10^0^	8.0 × 10^0^	1.1 × 10^1^

*The numbers of NoV genome copies per gram of oyster digestive gland were left uncorrected.

## Discussion

In recent years, NoV outbreaks have attracted increasing attention from public and researchers, and oysters are one of the main causes of the outbreaks (Cheng et al., 2005). Oysters are filter-feeding animals, and their digestive glands have substances similar to the NoVs receptor Human blood group antigens (HBGAs), which could help the virus be accumulated in the oysters (McLeod et al., 2009). Therefore, the digestive gland is used as the target tissue for the NoV detection in oysters in the ISO/TS 15216-2:2013 standard method.

IME pre-treatment methods have the advantage of being easy to use, not requiring complicated equipment, and achieving effective removal of inhibitors and specific enrichment of pathogens in the samples. In this study, the T-IME, SA-BA-IME, and P-SA-BA-IME methods were established and compared. The T-IME enrichment method has limitations (Wang et al., 2011). It is based on the coupling of antibodies with magnetic beads directly through covalent bonds, however, the surface of the magnetic beads is rigid, and the connection stability is extremely poor, which may lead to changes in the spatial structure of the antibody and reduce its activity (Shan et al., 2014). Changes in the spatial direction of antibodies increase the steric hindrance between them, which will also reduce the CE (Jo et al., 2015). SA-BA-IME combines magnetic bead-coupled SA and biotinylated antibodies, which improves the stability during the reaction and indirectly increases the area where the antibody molecule binds to the target, so a higher CE can be obtained. P-SA-BA-IME consists of a two-stage amplification based on SA one-stage amplification.

IMS methods have been applied for detecting NoVs in different food samples (Park et al., 2008; Lee et al., 2013; Ha et al., 2014), the antibodies are the main factor affecting the broad spectrum of enrichment methods. Park et al. used polyclonal antibody coupled with magnetic beads (280 μm) combined with real-time RT-PCR to detect GI.1 and GII.4 NoV in artificially contaminated strawberries, the polyclonal antibody used in this method can react with more than 31 NoV genotypes (Park et al., 2008). The maximum recovery rate of this method was 30% lower than that of P-SA-BA-IME, but it had a good broad-spectrum combination. It should be noted that GI NoVs are also widely detected in foods such as shellfish. However, the diversity of human NoVs poses a challenge for the simultaneous capture of GI and GII NoVs. The development of high-titer and broad-spectrum antibodies is the key to the development of the NoV enrichment method with broader spectrum in the future.

As the particle size of the magnetic beads decreased, the number of magnetic beads that could be bound to the pathogen surface increased significantly. Yang et al. (2007) studied the CE of nanomagnetic beads and micromagnetic beads, and the results showed that the capture efficiency of nanomagnetic beads was 1–2 times higher than that of micromagnetic beads. The lower concentration of the targets in the solution, the stronger capture ability of the nanomagnetic beads. Therefore, nanomagnetic beads were selected for this study instead of micromagnetic beads to improve the CE.

In this study, PAMAM was used as the coupling carrier of biotin to achieve biotin amplification. However, when the amount of biotin-mAb was excessive, it competed to bind to SA magnetic beads, resulting in a decrease in CE. The anti-NoV mAb used in this study was prepared by using GII.4 NoV capsid P protein as immunogen with the titer >10^6^, ELISA results showed that the anti-NoV mAb could react with GII.2, GII.3, GII.4, GII.6, GII.17 NoVs (Gao et al., 2021), which ensured the high efficiency of NoVs enrichment. The amount of biotin-mAb and the selection of anti-NoVs mAb are both the important factors affecting CE. And oysters purchased from the market were directly used for the evaluation of the three methods, the detection rate of the P-SA-BA-IME method was 25.0% which is higher than that of ISO/TS standard method for detection of retail oysters (20.71%, 16.9%) in China (Jiang et al., 2018; Zhang et al., 2021). However, we developed a modified ISO/TS 15216-2:2013 standard method which combined with the PEG precipitation concentration method to increase the detection rate to 34.21% (Zhang et al., 2020). In the next work, the sensitivity of P-SA-BA-IME method can be further improved by changing the antibody or optimizing the conditions. Few samples were tested negative by P-SA-BA-IME but positive by T-IME and SA-BA-IME, which may have been caused by the low viral load. Besides, the genome copies numbers in some oyster detected by the new method were also not the highest, this may be due to insufficient mixing when the sample is divided into three equal parts resulting in uneven distribution, or the influence of impurities, IMBs and the NoVs in the suspension did not fully bind during the limited incubation time. A large number of samples may reflect the advantages of the P-SA-BA-IME method. The results showed that the CE of the T-IME was 15.63 ± 0.43%, and the SA-BA-IME improved the CE to 37.48 ± 0.20%. P-SA-BA-IME efficiently binds SA magnetic beads and PAMAM-conjugated biotin-mAb, with a CE of 44.26 ± 1.45%.

In summary, we established a sensitive and efficient pre-treatment method for NoV detection in oysters. PAMAM-conjugated biotin-mAb combined with SA-biotin IMBs were used for the enrichment of NoVs in oysters for the first time, showing the utility of this method to increase the detection rate of NoVs in oysters. This is important for preventing NoV infection from shellfish.

## Data Availability Statement

The original contributions presented in the study are included in the article/Supplementary Material, further inquiries can be directed to the corresponding author/s.

## Author Contributions

JG, LX, and JZ designed the research. JG and LZ performed the experiments and wrote the manuscript. WC, ZQ, JY, YaL, and LW contributed to the reagents and materials. JG, LX, and WC analyzed the data. LX, JZ, and QW performed critical revisions of the manuscript. All authors contributed to the article and approved the submitted version.

## Conflict of Interest

The authors declare that the research was conducted in the absence of any commercial or financial relationships that could be construed as a potential conflict of interest.
