# Supplementary material for: Development of a High-Efficiency Immunomagnetic Enrichment Method for Detection of Human Norovirus via PAMAM Dendrimer/SA-Biotin Mediated Cascade-Amplification
Source: Front Microbiol. 2021 Jul 20;12:673872. doi: 10.3389/fmicb.2021.673872 (PMC8329424; doi:10.3389/fmicb.2021.673872)
Supplement: Supplementary file 1 [file Table_1.DOCX]

Table S1 Optimization of couple time of traditional immunomagnetic enrichment (T-IME)

| Couple time(h) | Capture efficiency (%) | | | | | $\bar{x}$ (%) | SD (%) |
| --- | --- | --- | --- | --- | --- | --- | --- |
|  | 1 | | 2 | | 3 |  |  |
| 0.5 | 6.87 | 6.67 | | 4.87 | | 6.14 | 0.90 |
| 1 | 9.53 | 10.51 | | 9.19 | | 9.74 | 1.56 |
| 1.5 | 13.85 | 11.40 | | 12.80 | | 12.68 | 1.00 |
| 2 | 13.39 | 13.30 | | 12.44 | | 13.04 | 0.43 |
| 2.5 | 13.94 | 12.66 | | 14.16 | | 13.59 | 0.66 |

Table S2 Optimization of the amount of immunomagnetic beads of traditional immunomagnetic enrichment (T-IME)

| Amount of IMB(mg) | Capture efficiency (%) | | | | | $\bar{x}$ (%) | SD (%) |
| --- | --- | --- | --- | --- | --- | --- | --- |
|  | 1 | | 2 | | 3 |  |  |
| 0.025 | 6.93 | 8.27 | | 5.59 | | 6.93 | 1.10 |
| 0.05 | 11.09 | 8.54 | | 11.27 | | 10.30 | 1.25 |
| 0.1 | 12.49 | 12.52 | | 12.72 | | 12.58 | 0.10 |
| 0.15 | 13.15 | 13.27 | | 13.24 | | 13.22 | 0.05 |
| 0.2 | 13.36 | 12.72 | | 13.04 | | 13.04 | 0.26 |

Table S3 Optimization of enrichment time of traditional immunomagnetic enrichment (T-IME)

| Enrichment time(min) | Capture efficiency (%) | | | | | $\bar{x}$ (%) | SD (%) |
| --- | --- | --- | --- | --- | --- | --- | --- |
|  | 1 | | 2 | | 3 |  |  |
| 15 | 5.55 | 6.35 | | 5.80 | | 5.90 | 0.33 |
| 30 | 7.62 | 7.23 | | 7.81 | | 7.55 | 0.24 |
| 45 | 9.23 | 9.94 | | 9.32 | | 9.50 | 0.32 |
| 60 | 15.38 | 14.68 | | 14.23 | | 14.76 | 0.48 |
| 75 | 15.18 | 14.74 | | 15.35 | | 15.09 | 0.26 |

Table S4 Optimization of separation time of traditional immunomagnetic enrichment (T-IME)

| Separation time(min) | Capture efficiency (%) | | | | | $\bar{x}$ (%) | SD (%) |
| --- | --- | --- | --- | --- | --- | --- | --- |
|  | 1 | 2 | | 3 | |  |  |
| 1 | 6.24 | | 7.84 | | 8.09 | 7.39 | 0.82 |
| 3 | 9.13 | | 8.15 | | 7.72 | 8.33 | 0.59 |
| 5 | 9.96 | | 10.56 | | 10.24 | 10.25 | 0.24 |
| 7 | 15.70 | | 15.08 | | 16.13 | 15.63 | 0.43 |
| 9 | 14.81 | | 15.28 | | 15.84 | 15.31 | 0.42 |

Table S5 Optimization of amount of biotin-mAb in streptavidin-biotin amplified immunomagnetic enrichment (SA-BA-IME)

| Amount of Biotin-mAb (mg) | Capture efficiency (%) | | | | | $\bar{x}$ (%) | SD (%) |
| --- | --- | --- | --- | --- | --- | --- | --- |
|  | 1 | | 2 | | 3 |  |  |
| 10 | 16.98 | 15.28 | | 16.38 | | 16.21 | 0.70 |
| 20 | 19.42 | 19.12 | | 19.90 | | 19.48 | 0.32 |
| 30 | 22.16 | 21.05 | | 24.07 | | 22.43 | 1.25 |
| 40 | 33.38 | 38.70 | | 40.74 | | 37.61 | 3.10 |
| 50 | 17.09 | 16.45 | | 17.28 | | 16.94 | 0.36 |

Table S6 Optimization of immunoreaction time of biotin-mAb against noroviruses in streptavidin-biotin amplified immunomagnetic enrichment (SA-BA-IME)

| Immunoreaction time(min) | Capture efficiency (%) | | | | | $\bar{x}$ (%) | SD (%) |
| --- | --- | --- | --- | --- | --- | --- | --- |
|  | 1 | 2 | | 3 | |  |  |
| 5 | 26.39 | | 25.52 | | 26.04 | 25.98 | 0.36 |
| 15 | 34.22 | | 31.64 | | 31.50 | 32.45 | 1.25 |
| 25 | 35.94 | | 36.68 | | 38.70 | 37.11 | 1.17 |
| 35 | 38.96 | | 36.27 | | 37.59 | 37.61 | 1.10 |
| 45 | 38.44 | | 37.09 | | 37.93 | 37.82 | 0.56 |

Table S7 Optimization of amount of immunomagnetic beads in streptavidin-biotin amplified immunomagnetic enrichment (SA-BA-IME)

| Amount of IMB(mg) | Capture efficiency (%) | | | | | $\bar{x}$ (%) | SD (%) |
| --- | --- | --- | --- | --- | --- | --- | --- |
|  | 1 | | 2 | | 3 |  |  |
| 40 | 22.21 | 21.53 | | 22.61 | | 22.12 | 0.45 |
| 60 | 31.78 | 33.68 | | 34.76 | | 33.41 | 1.23 |
| 80 | 37.67 | 37.93 | | 34.68 | | 36.76 | 1.48 |
| 100 | 37.67 | 38.18 | | 37.76 | | 37.87 | 0.22 |
| 120 | 37.42 | 37.01 | | 37.51 | | 37.31 | 0.22 |

Table S8 Optimization of incubation time of streptavidin-biotin amplified immunomagnetic enrichment (SA-BA-IME)

| Incubation time(min) | Capture efficiency (%) | | | | | $\bar{x}$ (%) | SD (%) |
| --- | --- | --- | --- | --- | --- | --- | --- |
|  | 1 | | 2 | | 3 |  |  |
| 10 | 28.10 | 26.99 | | 31.64 | | 28.91 | 1.98 |
| 20 | 30.12 | 33.16 | | 33.09 | | 32.12 | 1.42 |
| 30 | 37.76 | 37.34 | | 37.34 | | 37.48 | 0.20 |
| 40 | 36.43 | 36.43 | | 37.09 | | 36.65 | 0.31 |
| 50 | 38.35 | 36.19 | | 35.94 | | 36.83 | 1.08 |

Table S9 Optimization of amount of biotin-PAMAM-mAb in polyamidoamine (PAMAM) dendrimer/SA-biotin mediated cascade-amplification IME (P-SA-BA-IME)

| Amount of Biotin-PAMAM-mAb (mg) | Capture efficiency (%) | | | | | $\bar{x}$ (%) | SD (%) |
| --- | --- | --- | --- | --- | --- | --- | --- |
|  | 1 | | 2 | | 3 |  |  |
| 10 | 28.48 | 29.85 | | 25.18 | | 27.83 | 1.96 |
| 20 | 27.47 | 25.98 | | 31.28 | | 28.25 | 2.23 |
| 30 | 30.12 | 27.35 | | 34.68 | | 30.72 | 3.02 |
| 40 | 39.22 | 43.09 | | 38.35 | | 40.22 | 2.06 |
| 50 | 16.71 | 17.25 | | 17.75 | | 17.24 | 0.43 |

Table S10 Optimization of immunoreaction time of biotin-PAMAM-mAb with noroviruses in polyamidoamine (PAMAM) dendrimer/SA-biotin mediated cascade-amplification IME (P-SA-BA-IME)

| Immunoreaction time(min) | Capture efficiency (%) | | | | | $\bar{x}$ (%) | SD (%) |
| --- | --- | --- | --- | --- | --- | --- | --- |
|  | 1 | 2 | | 3 | |  |  |
| 5 | 30.87 | | 28.93 | | 33.91 | 31.23 | 2.05 |
| 15 | 41.76 | | 41.76 | | 41.39 | 41.64 | 0.18 |
| 25 | 40.93 | | 40.93 | | 39.05 | 40.30 | 0.89 |
| 35 | 39.49 | | 42.14 | | 40.02 | 40.55 | 1.14 |
| 45 | 41.39 | | 42.90 | | 40.74 | 41.68 | 0.90 |

Table S11 Optimization of the amount of immunomagnetic beads in polyamidoamine (PAMAM) dendrimer/SA-biotin mediated cascade-amplification IME (P-SA-BA-IME)

| Amount of IMB(mg) | Capture efficiency (%) | | | | | $\bar{x}$ (%) | SD (%) |
| --- | --- | --- | --- | --- | --- | --- | --- |
|  | 1 | | 2 | | 3 |  |  |
| 40 | 18.32 | 25.40 | | 28.16 | | 23.96 | 4.14 |
| 60 | 43.28 | 44.07 | | 39.93 | | 42.43 | 1.79 |
| 80 | 43.58 | 44.46 | | 36.76 | | 41.60 | 3.44 |
| 100 | 43.67 | 37.34 | | 44.07 | | 41.69 | 3.08 |
| 120 | 41.57 | 41.85 | | 41.20 | | 41.54 | 0.27 |

Table S12 Optimization of incubation time of in polyamidoamine (PAMAM) dendrimer/SA-biotin mediated cascade-amplification IME (P-SA-BA-IME)

| Incubation time(min) | Capture efficiency (%) | | | | | $\bar{x}$ (%) | SD (%) |
| --- | --- | --- | --- | --- | --- | --- | --- |
|  | 1 | | 2 | | 3 |  |  |
| 10 | 29.45 | 30.87 | | 32.50 | | 30.94 | 1.25 |
| 20 | 42.23 | 44.96 | | 45.57 | | 44.26 | 1.45 |
| 30 | 41.02 | 45.17 | | 44.96 | | 43.72 | 1.19 |
| 40 | 45.37 | 42.14 | | 41.48 | | 43.00 | 1.70 |
| 50 | 45.17 | 42.90 | | 43.09 | | 43.72 | 1.03 |
